# Pharmacological Proprieties of the Ethanol Extract of *Muehlenbeckia platyclada* (F. Muell.) Meisn. Leaves

**DOI:** 10.3390/ijms11103942

**Published:** 2010-10-15

**Authors:** Leopoldina Leonor Fagundes, Glauciemar Del-Vechio Vieira, José de Jesus R. G. de Pinho, Célia Hitomi Yamamoto, Maria Silvana Alves, Paulo César Stringheta, Orlando Vieira de Sousa

**Affiliations:** 1 Departamento Farmacêutico, Faculdade de Farmácia e Bioquímica, Universidade Federal de Juiz de Fora, Campus Universitário, Martelos, 36036-330, Juiz de Fora, MG, Brazil; E-Mails: dinafgs@hotmail.com (L.L.F.); glauciemar@gmail.com (G.D.-V.V.); jose.pinho@ufjf.edu.br (J.J.R.G.P.); hytomani@yahoo.com (C.H.Y.); 2 Departamento de Análises Clínicas, Faculdade de Farmácia e Bioquímica, Universidade Federal de Juiz de Fora, Campus Universitário, Martelos, 36036-330, Juiz de Fora, MG, Brazil; E-Mail: alves_ms2005@yahoo.com.br; 3 Departamento de Tecnologia de Alimentos, Centro de Ciências Exatas e Tecnológicas, Universidade Federal de Viçosa, Campus Universitário, 36571-000 -Viçosa, MG, Brazil; E-Mail: pstringheta@yahoo.com.br

**Keywords:** Muehlenbeckia platyclada, Polygonaceae, antinociceptive activity, anti-inflammatory activity

## Abstract

Antinociceptive and anti-inflammatory activities of the *Muehlenbeckia platyclada* leaves’ ethanol extract were investigated in animal models. The extract (p.o.) reduced the number of abdominal contortions induced by acetic acid by 21.57% (400 mg/kg). After intraplantar injection of formalin, a dose of 400 mg/kg (p.o.) inhibited the time spent paw licking in the first phase (26.43%), while the second phase was inhibited by 10.90 and 36.65% at the doses of 200 and 400 mg/kg, respectively. The extract (p.o.) increased the reaction time on a hot plate at a dose of 400 mg/kg (32.68 and 40.30%) after 60 and 90 minutes of treatment, respectively. The paw edema was reduced by extract (p.o.) at doses of 100 (15.46 and 16.67%), 200 (22.68 and 25.64%) and 400 mg/kg (29.50 and 37.33%) after 3 to 4 h of carrageenan application, respectively. Doses of 100, 200 and 400 mg/kg (p.o.), administered 4 h after the carrageenan injection, reduced the exudate volume (11.28, 21.54 and 45.13%), while leukocyte migration was reduced by 21.21 and 29.70% at the doses of 200 and 400 mg/kg, respectively. These results indicate that the ethanol extract from *M. platyclada* may constitute a potential target for the discovery of new molecules with antinociceptive and anti-inflammatory activities that can be explored for their therapeutic use.

## 1. Introduction

Medicinal plants are one of the most important sources of active substances with therapeutic potential and these are often used to cure a variety of diseases in humans [1,2]. Moreover, the evaluation of pharmacological effects can be used as a strategy for discovering new drugs of plant origin [2,3]. In particular, the use of medicinal plants as anti-inflammatory and analgesic agents is a common practice and have been the target of recent studies [4,5].

The genus *Muehlenbeckia* is constituted by four species belonging to the family Polygonaceae. Plants of this genus, such as *Muehlenbeckia platyclada* (F. Muell.) Meisn., commonly known as carqueja de jardim or fita de moça, are found in South America, and have been traditionally used as diuretic, hypotensive, antihemorragic, sedative, antirheumatic, abortive, cicatrizant, antiulcerogenic, anti-inflammatory and anthelmintic agents [6,7].

Among the active compounds found in *M. platyclada*, the flavonoids (morin-3-*O*-α-rhamnopyranoside, kaempferol-3-*O*-α-rhamnopyranoside, kaempferol-3-*O*-β-glucopyranoside, quercetin 3-*O*-α-rhamnopyranoside and (+)-catechin), were reported [8]. However, in species of *Muehlenbeckia*, such as *M. tamnifolia*, free anthraquinones (chrysophanic acid, emodin, and rhein) and glycoside anthraquinones have been identified [9]. Epicatechin, emodin-8-glycoside and rutin were identified from the aerial part and the root of *M. hastulata* [10]. Pheophorbide A, hypericin and protohypericin, isolated from this specie, demonstrated potent activity against influenza virus [11].

Considering pharmacological studies, *M. hastulata* possesses significant oxytoxic and analgesic activities [10]. The chloroform, butanol and aqueous extracts as well as flavonoids isolated from *M. platyclada* inhibited the generation of superoxide anion and elastase released by human neutrophils, indicating anti-inflammatory activity [8]. Based on the anthelmintic property, the methanolic extract of *M. platyclada* did not exhibit activity against *Bursaphelenchus xylophilus* [12].

In order to evaluate the pharmacological basis for a better understanding of the use of *M. platyclada* in folk medicine, the present study was designed to investigate the antinociceptive and anti-inflammatory effects of ethanol extract obtained from leaves using animal models.

## 2. Results and Discussion

### 2.1. Phytochemistry Screening

The phytochemical screening results of the ethanol extract showed the presence of different types of active constituents like flavonoids, terpenoids, sterols, coumarins, tannins, saponins and volatile oils.

### 2.2. Acute Toxicity

At the doses administered per oral route (p.o.), the ethanol extract from *M. platyclada* leaves was toxic to animals with LD_50_ of 2.67 g/kg (95% confidence intervals 1.65–4.31 g/kg). This result was applied as a parameter to establish the dosage definition in the experiments of pharmacological activities.

### 2.3. Writhing Response Induced by Acetic Acid in Mice

**Figure 1:**
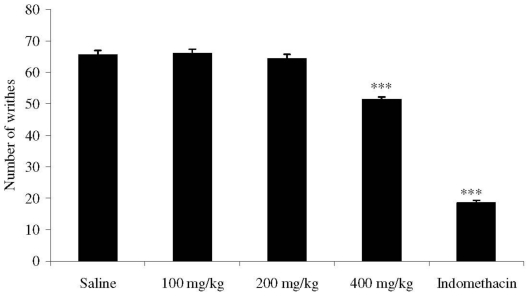
Effects of the ethanol extract from *M. platyclada* leaves on acetic acid-induced writhing in mice. Data are mean ± s.e.m. of eight mice.***P < 0.001 *vs.* control group.

Dose (p.o.) of 400 mg/kg of *M. platyclada* extract significantly reduced (*p* < 0.001) the abdominal contortions induced by acetic acid to 51.37 ± 0.84 compared to the respective control (65.50 ± 1.50) (Figure 1).

### 2.4. Effects on Formalin-Induced Nociception in Mice

**Figure 2:**
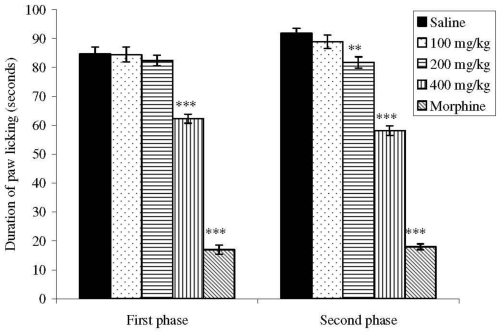
Effects of the ethanol extract from *M. platyclada* leaves on formalin-induced nociception in mice. First phase = 0–5 min after formalin injection; second phase = 15–30 min. Data are mean ± s.e.m. of eight mice. **P < 0.01; ***P < 0.001 *vs.* control group.

The intraplantar injection of formalin promoted a biphasic characteristic response (Figure 2). The time spent licking in the first phase (0–5 min) was 84.62 ± 2.45 s and in the second phase (15–30 min) was 91.75 ± 1.79 s for the control group. After 60 min of treatment, a dose (p.o.) of 400 mg/kg of extract significantly inhibited (*p* < 0.001) the first phase at 26.43% and the doses of 200 and 400 mg/kg reduced the second phase at 10.90 and 36.65%, respectively, when compared to the control.

### 2.5. Effects on Hot-Plate Latency Assay in Mice

The *M. platyclada* ethanol extract increased the latency time of mice exposed to the hot plate (Table 1). After 60 and 90 min of treatment, dose (p.o.) of 400 mg/kg (32.68 and 40.31%) increased significantly (*p* < 0.05 and *p* < 0.01, respectively) the latency time in the respective control group. Morphine proved to be a potent analgesic, increasing the latency time within the evaluation periods. Naloxone, an opioid antagonist, blocked the morphine action but did not alter the antinociceptive effect of the tested extracts.

### 2.6. Effects on Carrageenan-Induced Edema in Rats

The *M. platyclada* ethanol extract anti-inflammatory effect evaluated by the paw edema method induced by carrageenan is shown in Table 2. Edema inhibition was observed 3 h after carrageenan application of doses (p.o.) of 100 (0.82 ± 0.03; 1.46 %; *p* < 0.05), 200 (0.75 ± 0.04; 22.70 %; *p* < 0.01) and 400 mg/kg (0.63 ± 0.04; 35.05 %; *p* < 0.001). 4 h after carrageenan injections, the doses of 100 (0.65 ± 0.02; *p* < 0.05), 200 (0.58 ± 0.03; *p* < 0.01) and 400 mg/kg (0.52 ± 0.02; *p* < 0.001) reduced the respective paw edema (16.67, 25.64 and 33.33%). In this time, indomethacin also reduced the paw edema (39.74%).

### 2.7. Effects on Carrageenan-Induced Pleurisy in Rats

The pleurisy effects demonstrated that doses (p.o.) of 100 (*p* < 0.05), 200 (*p* < 0.01) and 400 mg/kg (*p* < 0.001) of the extracts significantly reduced the exudate volume (Table 3). The number of total leukocytes was inhibited at the doses of 200 (*p* < 0.001) and 400 mg/kg (*p* < 0.001) (Table 3). The exudate volume was decreased by 11.28, 21.54 and 45.13% at doses (p.o.) of 100, 200 and 400 mg/kg compared to the respective control. Leukocyte migration inhibition also occurred from doses (p.o.) of 200 (13.00 ± 0.21 × 10^3^ cells/mm^3^; *p* < 0.001) and 400 mg/kg (11.60 ± 0.32 × 10^3^ cells/mm^3^; *p* < 0.001). Indomethacin reduced the exudate volume and the leukocyte migration.

The acute toxicity test showed that the *M. platyclada* leaves’ ethanol extract doses tested were toxic to mice. However, the largest dose administered (400 mg/kg) is less than the lowest dose applied for determination of the LD_50_ (0.5 g/kg or 500 mg/kg). This is the first time that the toxic effect on plants of the genus *Muehlenbeckia* is described. Probably, the toxic effect of the ethanol extract is due to the presence of compounds, such as saponins [13,14]. In the present study, the LD_50_ was used to define the doses that were administered to the animals.

Considering the antinociceptive activity, the intraperitoneal administration of acetic acid induces the synthesis of the prostaglandins and sympathomimetic system mediators like PGE2 and PGF2α [15]. Thus, the antinociceptive effect of the ethanol extract could be due to the inhibition of these mediators. This effect was also demonstrated by the biphasic response time of paw licking induced by formalin [16]. The first phase (0 to 5 min) corresponds to the neurogenic stage as an intensely painful process for the activation of nociceptive pathways, while inflammation mediators are produced after 15 minutes of formalin application (second phase) [16,17]. Substance P and bradykinin act as mediators in the first phase, while histamine, serotonin, prostaglandin and bradykinin are involved in the nociceptive response of the second stage [17].

The central action was confirmed in the hot plate test (400 mg/kg), showing that the maximum effect was reached after 90 minutes. This test is considered to be sensitive to drugs acting at the supraspinal modulation level of the pain response [18], suggesting a possible modulatory effect of the extract. Furthermore, antinociceptive action was not dependent on the opioid system, because naloxone treatment did not reverse the produced effect [19,20].

The algesia test induced by formalin also indicated a possible anti-inflammatory activity (the second phase was reduced from 200 mg/kg). This activity was confirmed by the paw edema induced by carrageenan in rats, an animal model widely used to investigate anti-inflammatory substances. Carrageenan induces paw edema resulting in the release of mediators such as histamine, serotonin, bradykinin, substance P, platelet activating factor and prostaglandins [21–27]. Oral treatment with the *M. platyclada* ethanol extract significantly inhibited the paw edema from 100 mg/kg. This result suggests that the anti-inflammatory actions of the extract are related to inhibition of one or more signaling intracellular pathways involved with the release of mediators mentioned above.

The formation of exudate in the pleural cavity [28,29] and leukocyte migration [29,30] were produced by intrapleural injection of carrageenan and this method was used to confirm the obtained paw edema results. Non-steroidal anti-inflammatory drugs, such as indomethacin, inhibit the accumulation of exudates and mobilization of leukocytes between 3 and 6 h after application of carrageenan [29,31]. The ethanol extract from *M. platyclada* reduced the volume of exudate and the leukocyte migration, also corroborating the anti-inflammatory activity (Tables 2 and 3).

Our phytochemical screening demonstrated the presence of flavonoids, saponins, tannins, terpenoids, sterols, coumarins, volatile oils, triterpenes and steroids. Flavonoids, like morin-3-*O*-α-rhamnopyranoside, kaempferol-3-*O*-α-rhamnopyranoside, kaempferol-3-*O*-β-glucopyranoside, quercetin 3-*O*-α-rhamnopyranoside and (+)-catechin, have been isolated from *M. platyclada* [8]. These flavonoids inhibited the generation of superoxide anion and elastase released by human neutrophils, indicating anti-inflammatory activity [8]. Compounds like flavonoids [32] and triterpenes [33] have demonstrated anti-inflammatory activity, which may explain the antinociceptive activity of *M. platyclada*.

Several mechanisms of action could explain the pharmacological activities found in the present study. Flavonoids, for example, are potent inhibitors of nitric oxide synthase type 2 and protein tyrosine kinases are important enzymes involved in the NO/cGMP pathway [34]. Flavonoids also can stimulate NOS-2 via indirect inhibition of the COX and/or lipoxygenase pathways [35] and of the protein kinase C and/or L-arginine/NO pathways [36]. These pathways have been implicated in a series of molecular events related to the nociceptive [37] and inflammatory [32] processes. In addition, flavonoids have shown ability to block phospholipase A2 and phospholipase C, which are key enzymes in inflammation [38]. The ability of flavonoids and triterpenes to inhibit the nuclear factor-kappaB (NF-κB) could clarify the anti-inflammatory activity of the extract [39]. According to Suh *et al*. [40], the antinociceptive activity of saponin has been associated with the modulation of the GABA_A_, NMDA and non-NMDA receptors at the supraspinal level. These authors have also reported that the nonopioid-mediated saponin antinociception is modulated via activation of the descending serotonin and α2-adrenergic pathways. However, additional studies are necessary to establish the possible correlation between activities and chemical composition of *M. platyclada* to ensure the appropriate medicinal use of this plant.

## 3. Experimental Section

### 3.1. Plant Material and Extraction

The plant material used in this study was collected in Juiz de Fora, in the state of Minas Gerais, Brazil, in June 2008. The species was identified by Dr Fátima Regina Gonçalves Salimena and a voucher specimen (CESJ number 53055) was deposited in the Herbarium of the Universidade Federal de Juiz de Fora, Brazil. Dried and powdered leaves (726 g) were exhaustively extracted in 95% ethanol (2.5 L) by static maceration for three weeks at room temperature with renewal of solvent every two days. The ethanol extract was filtered and evaporated under a rotary evaporator at controlled temperature (50–60 °C). This material was placed in a desiccator with silica to yield 42.72 g. The dried extract was dissolved using 1% DMSO in normal saline for pharmacological studies.

### 3.2. Phytochemical Screening of the Ethanol Extract

The screening of chemical constituents was carried out with the ethanol extracts using chemical methods and thin-layer chromatography (TLC), according to the methodology suggested by Matos [41], including flavonoids, tannins, coumarins, alkaloids, saponins, terpenoids, steroids and volatile oils.

### 3.3. Chemicals

Drugs and reagents used in this study (and their sources) were as follows: acetic acid (Vetec Química Farm Ltda, Rio de Janeiro, RJ, Brazil), formaldehyde (Reagen Quimibrás Ind. Química S.A., Rio de Janeiro, RJ, Brazil), morphine hydrochloride (Merck Inc., Whitehouse Station, NJ, USA), naloxone and indomethacin (Sigma Chemical Co, St Louis, MI, U.S.).

### 3.4. Animals

Male Wistar rats (90–110 days) weighing 200–240 g and male Swiss albino mice (50–70 days) weighing 25–30 g were used in the experiments. The animals were provided by the Central Biotery of the Universidade Federal de Juiz de Fora. The animals were divided into groups and kept in plastic cages (47 × 34 × 18 cm) under a 12 h light/12 h dark cycle at room temperature (22 ± 2 ºC), with free access to Purina rations and water. Animal care and the experimental protocol followed the principles and guidelines suggested by the Brazilian College of Animal Experimentation (COBEA) and were approved by the local ethical committee.

### 3.5. Acute Toxicity

Groups of ten mice received oral doses of 0.5, 1, 1.5, 2 and 3 g/kg of ethanol extract from *M. platyclada*, while the control group received the vehicle (saline). The groups were observed for 48 h and mortality at end of this period was recorded for each group [42]. The LD_50_ (50% lethal dose) was determined by probit test using a log plot of percentage death *versus* dose [43]. The determination of LD_50_ served to define the doses used in experiments of pharmacological activities.

### 3.6. Acetic Acid-Induced Writhing Response in Mice

Antinociceptive activity was evaluated using the test of abdominal writhing induced by acetic acid in mice [44]. Animals were divided into groups of eight mice. Control mice received an i.p. injection of acetic acid 0.6% (0.25 mL) and 10 min later the writhes were counted over a period of 20 min. One group of mice received indomethacin (10 mg/kg) by the per oral route (p.o.) as a reference compound, and the other three groups received the extract at doses (p.o.) of 100, 200 and 400 mg/kg, 1 h before the acetic acid injection.

### 3.7. Formalin-Induced Nociception in Mice

Mice received subplantar injections of 20 μL 2.5% formalin (in 0.9% saline) and the time of paw licking (in seconds) was determined over 0–5 min (first phase -neurogenic) and 15–30 min (second phase -inflammatory) after formalin injection [16]. Animals (n = 8) were pretreated p.o. with extract (100, 200 or 400 mg/kg; 0.1 mL per 10 g body weight) or the reference compound, subcutaneous morphine (1 mg/kg), 1 h before administration of formalin. Control animals were treated with sterile saline (10 mL/kg).

### 3.8. Hot-Plate Latency Assay in Mice

Animals were placed on a hot-plate (Model LE 7406, Letica Scientific Instruments, Barcelona, Spain) heated at 55 ± 1 °C [45]. Three groups of mice (n = 8) were treated p.o. with ethanol extract (100, 200 or 400 mg/kg; 0.1 mL per 10 g body weight); the control group received sterile saline (10 mL/kg). Measurements were performed at time 0, 30, 60 and 90 min after drug administration, with a cut-off time of 40 s to avoid lesions to the animals’ paws. The effect of pretreatment with naloxone (1 mg/kg, subcutaneously) on the analgesia produced by the ethanol extract (400 mg/kg) was determined in a separate group of animals. Morphine (1 mg/kg, subcutaneously), in the absence and presence of naloxone treatment, was used as a reference.

### 3.9. Carrageenan-Induced Edema in Rats

Anti-inflammatory activity was assessed on the basis of inhibition of paw edema induced by the injection of 0.1 mL of 2% carrageenan (an edematogenic agent) into the subplantar region of the right hind paw of the rat [46]. Male Wistar rats were divided into groups of six animals which received p.o. doses of extract (100, 200 and 400 mg/kg; 0.1 mL per 10 g body weight), saline or indomethacin (10 mg/kg) 1 h before the injection of carrageenan. In the left paw, used as a control, 0.1 mL of sterile saline was injected. 1, 2, 3 and 4 h after injection of carrageenan, the measure of edema was made by the difference between the volume displaced by the right paw and the left paw using a plethysmometer (model LE 7500, Letica Scientific Instruments, Barcelona, Spain).

### 3.10. Carrageenan-Induced Pleurisy in Rats

Pleurisy was induced in male Wistar rats by intrapleural administration of 0.5 mL 2% carrageenan suspension in saline solution between the third and fifth ribs on the right side of the mediastinum [37]. Extract (100, 200 and 400 mg/kg), saline or indomethacin (10 mg/kg) p.o. were given 60 min before injection of the irritant. Animals were killed 4 h after carrageenan injection, and the skin and pectoral muscles were retracted. A longitudinal incision was made between the third and fifth ribs on each side of the mediastinum. The exudate was collected and transferred to a 15 mL conical centrifuge tube and the total volume determined. A 20 μL aliquot of the exudate was used to determine the total leucocyte count in Neubauer chambers.

### 3.11. Calculus and Statistical Analysis

Data are expressed as mean ± s.e.m. Statistical significance was determined by one-way analysis of variance followed by the Student–Newman–Keuls test. *P* values below 0.05 were considered significant. The percentage of inhibition was calculated by using

100-T×100/C(%) or T×100/C-100(%)

where C and T indicate non-treated (vehicle) and drug-treated, respectively.

## 4. Conclusions

The present study demonstrated that the ethanol extract from *M. platyclada* leaves possesses antinociceptive and anti-inflammatory activities that could be related to the synergistic activity of the bioactive compounds, particularly flavonoids, triterpenes, saponins, and steroids. The results support the folklore use of this plant, but phytochemical studies together with pharmacological and toxicological investigations are essential for complete understanding of the medicinal application.

## Figures and Tables

**Table 1:** Effects of the ethanol extract from *M. platyclada* leaves on the reaction time(s) of mice exposed to the hot-plate test.

Group	Dose (mg/kg)	Time after drug administration (seconds)			
		0 min	30 min	60 min	90 min
Control	Saline	5.25 ± 0.65	5.75 ± 0.59	6.12 ± 0.64	6.50 ± 0.57
	100	5.12 ± 0.48	6.00 ± 0.73	6.37 ± 0.46	6.62 ± 0.62
Ethanol Extract	200	5.37 ± 0.73	6.12 ± 0.74	6.50 ± 0.63	6.75 ± 0.59
	400	5.12 ± 0.83	6.37 ± 0.73	8.12 ± 0.40*	9.12 ± 0.51**
Morphine	1	5.50 ± 0.57	9.50 ± 0.82**	14.37 ± 1.10***	17.37 ± 0.86***
Naloxone + Morphine	1 + 1	5.37 ± 0.70	7.50 ± 0.68	7.50 ± 0.38	6.87 ± 0.48
Naloxone + Extract	1 + 400	5.25 ± 0.88	6.50 ± 0.73	7.87 ± 0,40*	8.50 ± 0.53*

Data are mean ± s.e.m. of eight mice.

* *P* < 0.05

** *P* < 0.01

***P < 0.001 *vs.* control group.

**Table 2:** Effects of the ethanol extract from *M. platyclada* leaves on carrageenan-induced paw edema in rats.

Group	Dose (mg/kg)	Volume of hind paw (mL)			
		1 h	2 h	3 h	4 h
Control	Saline	0.55 ± 0.04	0.70 ± 0.05	0.97 ± 0.05	0.78 ± 0.05
	100	0.55 ± 0.08	0.68 ± 0.07	0.82 ± 0.03*	0.65 ± 0.02*
Ethanol Extract	200	0.53 ± 0.08	0.67 ± 0.08	0.75 ± 0.04**	0.58 ± 0.03**
	400	0.52 ± 0.07	0.63 ± 0.07	0.63 ± 0.04***	0.52 ± 0.02***
Indomethacin	10	0.50 ± 0.09	0.60 ± 0.08	0.62 ± 0.05***	0.47 ± 0.02***

Data are mean ± s.e.m. of six rats.

* *P* < 0.05

** *P* < 0.01

*** *P* < 0.001 *vs.* control group.

**Table 3:** Effects of the ethanol extract from *M. platyclada* leaves on pleural exudation and number of leucocytes in carrageenan-induced pleurisy in rats.

Group	Dose (mg/kg)	Exudate volume (mL)	Inhibition (%)	Nº Leucocytes (× 10^3^ cells/mm^3^)	Inhibition (%)
Control	Saline	1.95 ± 0.08	-	16.50 ± 0.50	-
	100	1.73 ± 0.03*	11.28	15.70 ± 0.43	4.85
Ethanol Extract	200	1.53 ± 0.07**	21.54	13.00 ± 0.21***	21.21
	400	1.07 ± 0.07***	45.13	11.60 ± 0.32***	29.70
Indomethacin	10	0.92 ± 0.08***	52.82	10.10 ± 0.35***	38.80

Data are mean ± s.e.m. of six rats.

* *P* < 0.05

** *P* < 0.01

*** *P* < 0.001 *vs.* control group.

## References

[1] Kamboj VP Herbal medicine Curr. Sci 2000 78 35 39

[2] Samy RP Gopalakrishnakone P Therapeutic potential of plants as anti-microbials for drug discovery Evid. Based Complement. Alternat. Med 2008 5 1 12 18955349 10.1093/ecam/nen036 PMC2887332

[3] Patwardhan B Vaidya ADB Chorghade M Ayurved and natural products drug discovery Curr. Sci 2004 86 789 799

[4] Sousa OV Del-Vechio-Vieira G Pinho JJRG Yamamoto CH Alves MS Antinociceptive and anti-Inflammatory activities of the ethanol extract of *Annona muricata* L. leaves in animal models Int. J. Mol. Sci 2010 11 2067 2078 20559502 10.3390/ijms11052067 PMC2885094

[5] Zakaria ZA Patahuddin H Mohamad AS Israf DA Sulaiman MR *In vivo* anti-nociceptive and anti-inflammatory activities of the aqueous extract of the leaves of *Piper sarmentosum* J. Ethnopharmacol 2010 128 42 48 20035852 10.1016/j.jep.2009.12.021

[6] Houghton PJ Manby J Medicinal plants of the Mapuche J. Ethnopharmacol 1985 13 89 103 3990317 10.1016/0378-8741(85)90063-7

[7] Villegas LF Fernfindez ID Maldonado H Torres R Zavaleta A Vaisberg AJ Hammond G Evaluation of the wound-healing activity of selected traditional medicinal plants from Perú J. Ethnopharmacol 1997 55 193 200 9080340 10.1016/s0378-8741(96)01500-0

[8] Yen CT Hsieh PW Hwang TL Lan YH Chang FR Wu YC Flavonol glycosides from *Muehlenbeckia platyclada* and their anti-inflammatory activity Chem. Pharm. Bull 2009 57 280 282 19252320 10.1248/cpb.57.280

[9] Martinod P Garcia L Hidalgo J Guevara C Anthraquinone pigments in *Muehlenbeckia tamnifolia* and *Muehlenbeckia vuleanica* Politécnica 1973 3 111 122

[10] Erazo S Muñoz O García R Lemus I Backhouse N Negrete R San Feliciano A Delporte C Constituents and biological activities from *Muehlenbeckia hastulata* Z. Naturforsch 2002 57c 801 804 10.1515/znc-2002-9-1008 12440715

[11] Yasuda T Yamaki M Iimura A Shimotai Y Shimizu K Noshita T Funayama S Antiinfluenza virus principles from *Muehlenbeckia hastulata* J. Nat. Med 2010 64 206 211 20082146 10.1007/s11418-009-0386-9

[12] Mackeen MM Ali AM Abdullah MA Nasir RM Mat NB Razak AR Kawazu K Antinematodal activity of some malaysian plant extracts against the pine wood nematode, *Bursaphelenchus xylophilus* Pestic. Sci 1997 51 165 170

[13] Francis G Kerem Z Makkar HPS Becker K The biological action of saponins in animal systems: A review Brit. J. Nutr 2002 88 587 605 12493081 10.1079/BJN2002725

[14] Melzig MF Hebestreit P Gaidi G Lacaille-Dubois MA Structure-activity-relationship of saponins to enhance toxic effects of agrostin Planta Med 2005 71 1088 1090 16320219 10.1055/s-2005-873112

[15] Deraedt R Jouquey S Delevallée F Flahaut M Release of prostaglandins E and F in an algogenic reaction and its inhibition Eur. J. Pharmacol 1980 51 17 24 7353582 10.1016/0014-2999(80)90377-5

[16] Hunskaar S Hole K The formalin test in mice: Dissociation between inflammatory and noninflammatory pain Pain 1987 30 103 114 3614974 10.1016/0304-3959(87)90088-1

[17] Shibata M Ohkubo T Takahashi H Inoki R Modified formalin test; characteristic biphasic pain response Pain 1989 38 347 352 2478947 10.1016/0304-3959(89)90222-4

[18] Yaksh TL Rudy TA Studies on direct spinal action of narcotics in production of analgesia in rat J. Pharmacol. Exp. Ther 1977 202 411 428 18600

[19] Sousa OV Del-Vechio-Vieira G Amaral MPH Pinho JJRG Yamamoto CH Alves MS Efeitos antinociceptivo e antiinflamatório do extrato etanólico das folhas de *Duguetia lanceolata* St. Hil. (Annonaceae) Lat. Am. J. Pharm 2008 27 398 402

[20] Silvério MS Sousa OV Del-Vechio-Vieira G Miranda MA Matheus FC Kaplan MAC Propriedades farmacológicas do extrato etanólico de *Eremanthus erythropappus* (DC.) McLeisch (Asteraceae) Rev. Bras. Farmacog 2008 18 430 435

[21] Di Rosa M Giroud JP Willoughby DA Studies on the mediators of the acute inflammatory response induced in rats in different sites by carrageenan and turpentine J. Pathol 1971 104 15 29 4398139 10.1002/path.1711040103

[22] Seibert K Zhang Y Leahy K Hauser S Masferrer J Perkins W Lee L Isakson P Pharmacological and biochemical demonstration of the role of cyclooxygenase 2 in inflammation and pain Proc. Natl. Acad. Sci. USA 1994 91 12013 12017 7991575 10.1073/pnas.91.25.12013 PMC45366

[23] Nantel F Denis D Gordon R Northey A Cirino M Metters KM Chan CC Distribution and regulation of cyclooxygenase-2 in carrageenan-induced inflammation Br. J. Pharmacol 1999 128 853 859 10556918 10.1038/sj.bjp.0702866 PMC1571708

[24] Stochla K Maślinśki S Carrageenan-induced oedema in the rat paw-histamine participation Agent. Act 1982 12 201 202 10.1007/BF01965145 6123241

[25] Hwang SB Lam MH Li CL Shen TY Release of platelet activation factor and its involvement in the first phase of carrageenin-induced rat foot edema Eur. J. Pharmacol 1986 120 33 41 3948914 10.1016/0014-2999(86)90636-9

[26] De Campos RO Alves RV Kyle DJ Chakravarty S Mavunkel BJ Calixto JB Antioedematogenic and antinociceptive actions of NPC 18521, a novel bradykinin B2 receptor antagonist Eur. J. Pharmacol 1996 316 277 286 8982699 10.1016/s0014-2999(96)00661-9

[27] Gilligan JP Lovato SJ Erion MD Jeng AY Modulation of carrageenan-induced hind paw edema by substance P Inflammation 1994 18 285 292 7522223 10.1007/BF01534269

[28] Ammendola G Di Rosa M Sorrentino L Leucocyte migration and lysosomal enzymes release in rat carrageenin pleurisy Agents Actions 1975 5 250 255 1236640 10.1007/BF02026439

[29] Almeida AP Bayer BM Horakova Z Beaven MA Influence of indomethacin and other anti-inflammatory drugs on mobilization and production of neutrophils: Studies with carrageenan induced inflammation in rats J. Pharmacol. Exp. Ther 1980 214 74 79 7391973

[30] Capasso F Dunn CJ Yamamoto S Willoughby DA Giroud JP Further studies on carrageenan-induced pleurisy in rats J. Pathol 1975 116 117 124 168331 10.1002/path.1711160208

[31] Vinegar R Truax JF Selph JL Some quantitative temporal characteristics of carrageenin induced pleurisy in the rat Proc. Soc. Exp. Biol. Med 1973 143 711 714 4719457 10.3181/00379727-143-37397

[32] Kim HP Son KH Chang HW Kang SS Antiinflammatory plant flavonoids and cellular action mechanisms J. Pharmacol. Sci 2004 96 229 245 15539763 10.1254/jphs.crj04003x

[33] Beirith A Santos ARS Calixto JB Hess SC Messana I Ferrari F Yunes RA Study of the antinociceptive action of the ethanolic extract and the triterpene 24-hydroxytormentic acid isolated from the stem bark of *Ocotea suaveolens* Planta Med 1999 65 50 55 10083846 10.1055/s-1999-13962

[34] Olszanecki R Gêbska A Kozlovski VI Gryglewski RJ Flavonoids and nitric oxide synthase J. Physiol. Pharmacol 2002 53 571 584 12512693

[35] Robak J Shridi F Wolbis M Krolikowska M Screening of the influence of flavonoids on lipoxygenase and cyclooxygenase activity, as well as on nonenzymic lipid oxidation Pol. J. Pharmacol. Pharm 1998 40 451 458 3151014

[36] Meotti FC Luiz AP Pizzolatti MG Kassuya CAL Calixto JB Santos ARS Analysis of the antinociceptive effect of the flavonoid myricitrin. Evidence for a role of the l-argininenitric oxide and protein kinase C pathways J. Pharmacol. Exp. Ther 2005 316 789 796 16260583 10.1124/jpet.105.092825

[37] Machelska H Labuz D Przewlocki R Przewlocka B Inhibition of nitric oxide synthase enhances antinociception mediated by mu, delta and kappa opioid receptors in acute and prolonged pain in the rat spinal cord J. Pharmacol. Exp. Ther 1997 282 977 984 9262366

[38] Middleton E Jr Kandaswami C Theoharides TC The effects of plant flavonoids on mammalian cells: Implications for inflammation, heart disease, and cancer Pharmacol. Rev 2000 52 673 751 11121513

[39] Nam NH Naturally occurring NF-kappaB inhibitors Mini Rev. Med. Chem 2006 6 945 951 16918500 10.2174/138955706777934937

[40] Suh HW Song DK Son KH Wie MB Lee KH Jung KY Do JC Kim YH Antinociceptive mechanisms of dipsacus saponin C administered intracerebroventricularly in the mouse Gen. Pharmacol 1996 27 1167 1172 8981063 10.1016/s0306-3623(96)00052-3

[41] Matos FJA Introdução à Fitoquímica Experimental2nd ed Edições UFC Fortaleza, Brazil 1997 41 75

[42] Dietrich L A new approach to practical acute toxicity testing Arch. Toxicol 1983 54 275 287 6667118 10.1007/BF01234480

[43] Litchfield JT Wilcoxon F A simplified method of evaluating dose-effect experiments J. Pharmacol. Exp. Ther 1949 96 99 113 18152921

[44] Collier HDJ Dinnin LC Johnson CA Schneider C The abdominal response and its suppression by analgesic drugs in the mouse Br. J. Pharmacol. Chemother 1968 32 295 310 4230818 10.1111/j.1476-5381.1968.tb00973.x PMC1570212

[45] Eddy NB Leimbach D Synthetic analgesics. II. Dithienylbutenyl and dithienylbutilamines J. Pharmacol. Exp. Ther 1953 107 385 393 13035677

[46] Winter CA Risley EA Nuss GW Carrageenin-induced edema in hind paw of the rat as an assay for anti-inflammatory drugs Proc. Soc. Exp. Biol. Med 1962 111 544 547 14001233 10.3181/00379727-111-27849

